# Outcome following radical cystectomy and bladder-preservation therapy in patients with invasive carcinoma of urinary bladder

**DOI:** 10.4103/0970-1591.38603

**Published:** 2008

**Authors:** B. S. Yadav, S. Ghoshal, S. C. Sharma

**Affiliations:** Department of Radiotherapy, PGIMER, Chandigarh, India

**Keywords:** Carcinoma, chemoradiation, radiotherapy, urinary bladder

## Abstract

**Background:**

Invasive bladder cancer is a lethal disease with a 50% cancer-related mortality even in the best healthcare systems. Optimum combination of surgery, external beam radiotherapy and platinum-based chemotherapy has yet to be determined.

**Purpose:**

To audit the outcome of multi-modality treatment and compare this with the existing literature in order to set future priorities and re-audit in patients with invasive carcinoma of urinary bladder.

**Materials and Methods:**

Between January 2001 and December 2004, 97 patients with invasive carcinoma of urinary bladder were analyzed. Radical surgery was done in 18(18%) patients and adjuvant radiation was given to 20(21%) patients. Radical radiation alone, (≥50 Gy) was given to 26(27%) and chemoradiation to 33(34%) patients respectively. Patients in the chemoradiation arm were given the same dose of radiation with weekly concomitant cisplatin at 40 mg/ m^2^ one hour before radiation during the first phase only. At a median follow-up of 32 months the outcome studied included locoregional failure, distant failure, disease-free survival (DFS) and overall survival (OS) using univariate and multivariate analyses. The OS and DFS were calculated according to Kaplan-Meier. Log rank test was used for statistical significance.

**Results:**

Median age of the patients was 58 years. Males comprised 93% of the total patients. Most (93%) of the patients had transitional cell histology. In patients treated with radiation alone overall response rate was 60%, with a complete response (CR) rate of 42%. The CR in patients treated with chemoradiation was 51%. Bladder was preserved in 61% of patients who received chemoradiation as compared to 42% in patients treated with radical radiation. With radical radiation local recurrence rate was 19% as compared to 22% with surgery and 6% with chemoradiation, respectively. Local recurrence rate was only 5% in patients treated with adjuvant radiation. Distant metastasis rate was least with chemoradiation (9%) as compared to 11.5% in radical radiation: curable dose of radiation and 33% with surgery alone, respectively. Patients with adjuvant radiation had a distant metastases rate of 15%. Median OS was 36 months. Factors affecting OS were histology (*P* = 0.023) and nodal involvement (*P* = 0.034). Median DFS was 26 months. Significant factors affecting DFS on univariate analysis were histology (*P* = 0.046) and nodal involvement (*P* = 0.004). On multivariate analysis the only factor affecting DFS and OS was nodal involvement (P = 0.01; Hazard Ratio, 0.085-0.719).

**Conclusion:**

In patients with invasive bladder cancer, combined modality in the form of radical cystectomy followed by radiation give best local control. Radiation alone is not effective to control muscle-invasive local disease; however, Chemoradiation is an effective alternative to radical cystectomy to preserve bladder function.

Bladder cancer includes a wide spectrum of diseases that can be divided into three major categories: superficial (70%), muscle-invasive (25%) and metastatic (5-10%). Tumor presentations differ in clinical behavior and they must be taken into account when determining prognosis and primary treatment. Five-year survival rate in muscle-invasive bladder cancer is 30-50%.[1] Treatment options for invasive cancer are: surgery, radical radiation and chemoradiation. A policy of radical radiotherapy or chemoradiation with surgical salvage produces the same survival rates as immediate surgery and allows a proportion of patients to retain their bladder.[2] Combination chemotherapy along with radiation therapy can also be used in patients who refuse surgery or are medically unfit for surgery. This retrospective analysis was done to analyze outcome in patients with invasive urinary bladder cancer in terms of locoregional failure, distant failure, disease-free survival (DFS) and overall survival (OS).

## MATERIALS AND METHODS

### Identification of patients

From January 2001 to December 2004, a total of 104 patients of invasive carcinoma of urinary bladder were retrospectively analyzed. Patients included were muscle-invasive carcinoma of urinary bladder (T2), serosal involvement (T3) and tumor involving prostrate, uterus and vagina (T4a) on histopathological examination or CT scan of the abdomen and pelvis. Pelvic lymph node metastases (detected by CT scan or ultrasound) were not considered exclusion criteria for surgery or other radical treatment. Patients' choice for a particular treatment was also given due consideration. Patients were classified in to four groups: radical cystectomy, adjuvant radiation, radical radiation and chemoradiation.

### Data collection and follow-up

Patients' information from the files was entered into a computer Excel sheet. Information collected included age, sex, presenting symptoms, radiological findings, procedure used for histopathological diagnosis, histology type, grade, tumor stage, treatment given (surgery, radiotherapy, chemoradiation or combined), complications of treatment and outcome. Lymphovascular emboli were defined from transurethral resection of bladder tumor (TURBT) or cystectomy specimen. Nodal involvement was defined from CT scan in patients who were not operated. Patients were kept on follow-up every two months till six months, four-monthly till two years and six-monthly thereafter. On every follow-up patients were evaluated by history, physical examination and any indicative investigation.

### Diagnosis and staging

Diagnosis was established with the help of TURBT in all patients. Routine clinical staging included a chest X-ray and abdominal and pelvic CT scan. Tumor category and grading were done according to TNM: tumor, node, metastases classification of 2002 (UICC) after going through surgical findings and histopathological reports.

### Surgical technique

In men, the standard surgical procedure was a cystoprostatectomy with pelvic lymphadenectomy. In women with T2 to T3a tumors, the standard surgical procedure was radical cystectomy with pelvic lymphadenectomy. Radical cystectomy in women also involved removal of the uterus, ovaries, fallopian tubes, anterior vaginal wall and urethra. The lymph node dissection was initiated 2 cm above the aortic bifurcation and extended laterally over the superior vena cava to the genitofemoral nerve. Distally, the lymph node dissection extended to the lymph node of Cloquet medially and the circumflex iliac vein laterally.

### Radiotherapy technique

Radiotherapy was initiated four to six weeks after TURBT. Dose given was 40 Gy/20#/4 weeks to pelvis by antero-posterior fields followed by 20 Gy/10#/2 weeks boost to bladder with 2 cm margins with three-field technique, one anterior and two posterior oblique fields in order to spare the spinal cord and rectum. Field borders for Phase I treatment were, upper border at the level of L5-S1 junction, lower border at inferior border of obturator foramen and lateral borders 1.5-2 cm from bony pelvis to include the pelvic lymph nodes. All patients were planned in the simulator in supine position. Patients in the chemoradiation arm were given the same dose of radiation with weekly concomitant cisplatin at 40 mg/m^2^ one hour before radiation during the first phase only. Treatment was delivered with cobalt-60 or 6 MV linear accelerator, all fields were treated daily. Response was assessed at six weeks of completion of therapy with cystocopy ± CT scan.

### Statistical analysis

The OS and DFS were calculated according to Kaplan-Meier. The OS was defined as from date of diagnosis till death or last follow-up. The DFS was defined from date of completion of treatment till any evidence of local or distant relapse. Log rank test was used for statistical significance. Multivariate analysis was performed by Cox regression model. A *P* value of < 0.05 was considered significant. All statistical analyses were conducted with the statistical software package SPSS version 12.00.

## RESULTS

**Figure 1:**
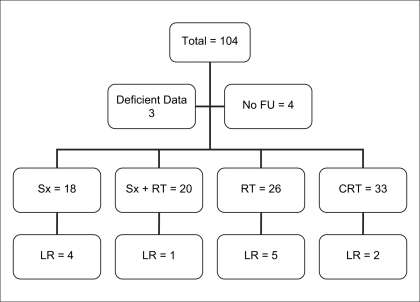
Flowchart showing different treatment groups

Patient characteristics are as shown in Table 1. Median age of the patients was 58 years (range 25-88 years). Males comprised 93% of the total patients. The majority 90(93%) of the patients had transitional cell carcinoma. Grade III/IV disease was seen in 64(66%) patients. Lymphovascular emboli were seen in 10(10%) patients and 20(21%) patients had pelvic lymph node metastases. Radical surgery was done in 18(18%) patients and adjuvant radiation was given to 20(21%) patients [Figure 1]. Radical radiation (≥50 Gy) was given to 26(27%) and chemoradiation to 33(34%) patients respectively.

**Table 1:** Patient characteristics

Characteristics	No (%)
Age (Median: 58 years)	
<60 years	51(53)
>60 years	46(47)
Sex	
Male	90(93)
Female	7(7)
Histology	
TCC	90(93)
SCC	4(4)
Adenocarcinoma	3(3)
Grade	
I and II	33(34)
III and IV	64(66)
LVE	
Present	10(10)
Absent	87(90)
Tumor	
T1 and T2	65(67)
T3 and T4	32(33)
Node	
N0	77(79)
N1	20(21)
Treatment	
Radical cystectomy	18(18)
Surgery + Radiation	20(21)
Radiation alone	26(27)
Chemoradiation	33(34)

Of 104 patients 97 were evaluable. Incomplete information in three patients and four patients were lost to follow-up after treatment. Transurethral resection of bladder tumor was done in all patients. Overstaging was found in two (11%) patients and understaging was seen in one (5.5%) patient after cystectomy. In patients treated with radiation alone overall response rates were 60%, with a CR rate of 42%. In patients treated with chemoradiation overall response rate was 72% with a CR of 51%. Bladder was preserved in 61% of patients who received chemoradiation as compared to 42% in patients treated with radical radiation alone.

With radical radiation local recurrence rate was 19% as compared to 22% with surgery and 6% with chemoradiation respectively. In patients treated with radical cystectomy followed by adjuvant radiation local recurrence rate was only 5%. A total of six patients were salvaged with radical cystectomy and two with partial cystectomy in the chemoradiation group. One in each salvage surgery developed distant metastases. Two patients treated with radical radiation were salvaged with radical cystectomy; both were disease-free at last follow-up. Most of the local recurrences (71%) with in the bladder occurred within three years. Distant metastasis rate was least with chemoradiation (9%) as compared to 11.5% in radiation alone and 33.3% with surgery alone respectively. Patients with adjuvant radiation had distant metastases rate of 15% i.e. it was reduced to half. This can be due to the fact that 10 (55.5%) of the patients with radical cystectomy had extravesical spread and four (22%) had positive pelvic lymph nodes as compared to seven (35%) extravesical spread and 11 (55%) positive pelvic nodes in patients treated with adjuvant radiation [Table 2]. Commonest site of distant metastases was liver (7%) followed by lungs (5%) and both in 5% of patients. It was observed that 83% of all the recurrences, local as well as distant occurred within two years after completion of treatment.

**Table 2:** Treatment group with high-risk features

Treatment	No.	Perivesical spread	Node positive	LVE	LR	Distant
Surgery	18	10	4	3	2	6
Surgery + RT	20	4	11	6	0	1
RT	26	8	2	0	3	1
CRT	33	7	3	1	3	1

Abbreviations: LVE, lymphovascular emboli; LR, local recurrence; RT, radiotherapy; CRT, chemoradiation

**Figure 2:**
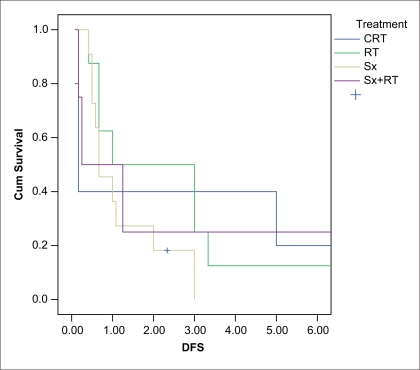
3 year DFS

**Figure 3:**
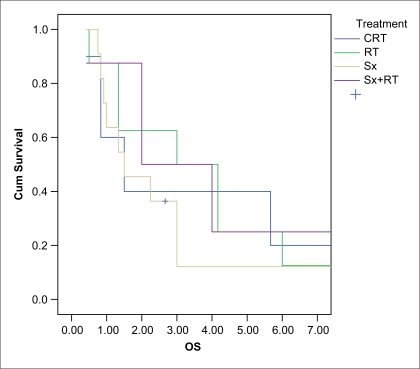
3 year overall survival

Median follow-up was 32 months. At last follow-up 68% of patients were without any evidence of disease, 28% were alive with disease, of which 13% had stable disease and 15% had progressive disease. Only 4% of the patients died of disease. Three-year DFS was 27% in the surgery and radiation group as compared to 50% in radiation alone and 40% in chemoradiation respectively [Figure 2]. This was not statistically significant (*P* = 0.52). Median DFS was 26 months. Significant factors affecting DFS on univariate analysis were histology (*P* = 0.046) and nodal involvement (*P* = 0.004). Median OS was 36 months. Three-year OS was 40% in chemoradiation, 38% in surgery alone group, 50% in surgery followed by radiation and radiation alone respectively [Figure 3]. This difference between the groups was not statiscally significant (*P* = 0.074). On univariate analysis factors affecting OS were histology (*P* = 0.023) and nodal involvement (*P* = 0.034). On multivariate analysis the only factor affecting DFS and OS was nodal involvement (*P* = 0.01; HR, 0.085-0.719).

Full doses of chemotherapy (four cycles) were tolerated by 54% of patients and 75% (three cycles) of chemotherapy were tolerated by 68% of patients. Only four patients had derangement in renal function, two of these patients had preexisting hydroureteronephrosis. Only two patients had Grade 2 hematological toxicity. Grade 3 acute rectal and bladder complications were seen in 2.5% and 3.5% of patients respectively. There were no treatment interruptions due to acute toxicities. A few patients had transient gastrointestinal symptoms, which were managed with medication. Only one patient had late toxicity in the form of hemorrhagic cystitis, it was managed conservatively. There was none severe late toxicity to rectum or small bowel. There was no treatment-related mortality.

## DISCUSSION

In the present analysis, we selected patients having invasive bladder cancer (T2, T3 and T4a). Local recurrences were least with radical cystectomy followed by adjuvant radiation; it was comparable to chemoradiation (5% *vs.* 6%). Radical cystectomy alone cannot prevent development of distant metastases. Distant metastasis rate was 33.3% after radical cystectomy alone as compared to 15% in patients treated with radical cystectomy followed by adjuvant radiation, i.e. it was reduced to half. This can be due to the fact that 10(55.5%) of the patients with radical cystectomy had extravesical spread and four(22%) had positive pelvic lymph nodes as compared to seven(35%) extravesical spread and 11(55%) positive pelvic nodes in patients treated with adjuvant radiation as shown in Table 2.

The primary treatment goal in oncology is to cure the patient, but at the same time maintaining function and quality of life is also important. Radical cystectomy is considered the standard of care for high-grade muscle-invasive bladder cancer. This treatment gives excellent survival rates and low incidence of local recurrence.[3] Definitive radiotherapy has been frequently used for preservation of bladder in cases of muscle-invasive bladder cancer but the results are not satisfactory. Overall survival at five years and local control rate is 22-35% and 35-40% respectively.[4] Radiation therapy achieves twice the rate of bladder preservation (40%) as compared to TURBT[5] (20%). Several studies have shown that patients treated with chemoradiation initially and cystectomy reserved for local failures, 40-80% of patients were able to retain their bladder function.[6–9]

Pelvic radiation in high-risk patients reduces local recurrence as well as distant metastases. The only significant factor affecting DFS and OS in this series was nodal metastases. Nodal metastases were more prevalent in patients treated with adjuvant radiation but none recurred locally and only one patient developed distant metastases. This means that adjuvant radiation controls local disease and further reduces chances of distant metastases. Although the total numbers of patients analyzed are small in each group, both radiation and chemoradiation seem to be equally effective in these patients with high-risk features [Table 2]. Pelvic lymph node metastases after radical cystectomy and lymphadenectomy have been pathologically reported in 11-17% of T2 and in 23-35% of T3 tumors, respectively.[10,11]

Locally advanced and muscle-invasive tumors are difficult to control, reported in previous studies of selective bladder-preserving therapy; 11-48% of the patients who had achieved CR at the time of tumor reevaluation after initial therapy had recurrence of invasive tumors.[12–15] This is because CR at reevaluation assures no residual tumor in part of the bladder mucosa; it is unknown whether there is residual tumor in the muscle layer of the bladder and perivesical tissue or it may be that tumor area is missed during cystoscopic reevaluation. In patients with T3 tumor treated with radical cystectomy, 2/3 of them developed distant metastases within two years of surgery. Similar findings were also reported by Hussain *et al*.[16]

Prognosis of patients with distant metastases from urinary bladder is very poor, with a median survival time of two years or less. The risk of distant metastases is associated with the extent of bladder wall invasion. Therefore prevention of distant metastases is essential to prolong survival. This can be done with adjuvant radiation to pelvis after radical cystectomy in high-risk patients; as seen in the present series none of the patients with high-risk features had local recurrence after adjuvant radiotherapy and only one patient had distant metastases. In patients with only radical cystectomy two had local recurrence and four had distant metastases within nine months. In such high-risk patients treated with chemoradiation distant metastases occurred in only one patient in spite of chemotherapy being used in radio-sensitizing doses only. Similar findings were reported by Hata *et al*.,[17] in a recent trial where they treated 25 patients with intra-arterial chemotherapy with methotrexate (30 mg/m^2^) and cisplatin (50 mg/m^2^) per course combined with proton therapy. The distant metastases rate was only 13%, facilitating 96% bladder preservation rate at five years. In Stage III patients who are not willing or are unfit to undergo radical cystectomy, definitive radiation therapy is an option that yields a five-year survival of approximately 30%.[18] The three-year survival rates in our series for patients treated with adjuvant radiation, radiation alone and chemoradiation are comparable to that reported in the literature[16] for patients with radical surgery. Surgery alone is not the optimal treatment for patients with Stage III bladder cancer. Chemoradiation appears to be best in the present scenario. It also makes bladder preservation feasible. Bladder preservation was possible in 61% of patients in the present series and is quite acceptable even though 49% of the treated patients have Stage III disease. Chemoradiation (with chemotherapy in full doses) or chemotherapy even during the boost irradiation of the bladder needs to be worked out.

Acute hematological toxicities due to chemoradiation were transient and gastrointestinal symptoms were mild. Late-term toxicities were not observed in this series. Our data is limited because of the small number of patients and retrospective nature of analysis; additional numbers of patients are needed to establish concrete results. This is a retrospective case series and the resultant bias prevents any firm conclusions to be drawn but the data do suggest areas for further study to formulate local guidelines. Still in our country we have to depend on this data to establish treatment because of different patient population and socioeconomic factors as compared to the western world. Unfortunately, primary cystectomy has not yet been tested against combined modality bladder-sparing treatment in randomized trials. If the comparison is restricted to the respective groups from the retrospective analyses, the five-year overall survival rates for radical cystectomy and bladder-sparing approaches become equal: 74 and 75%, respectively, for T1 tumors and 47 and 45%, respectively, for muscle-invasive disease.[19]

## CONCLUSION

Combined modality in the form of chemoradiation gives similar local control as surgery with the added advantage of bladder preservation. Nodal involvement affects both DFS and OS. Radiation alone is not effective to control muscle-invasive local disease; chemoradiation is another alternative with transient acute toxicities. Chemoradiation further reduces chances of distant metastases. It will be difficult to draw any firm conclusion from this small data as it is retrospective, non-randomized and with non-comparable arms. High-quality randomized controlled trials should be designed for confirmation. However, patient selection plays an important role in therapeutic outcome.
